# Prophylaxis for *Pneumocystis* pneumonia in patients with rheumatoid arthritis treated with biologics, based on risk factors found in a retrospective study

**DOI:** 10.1186/ar4472

**Published:** 2014-02-05

**Authors:** Takayuki Katsuyama, Kazuyoshi Saito, Satoshi Kubo, Masao Nawata, Yoshiya Tanaka

**Affiliations:** 1 First Department of Internal Medicine, School of Medicine, University of Occupational & Environmental Health, 1-1 Iseigaoka, Yahata-nishi, Kitakyushu 807-8555, Japan

## Abstract

**Introduction:**

*Pneumocystis* pneumonia (PCP) is one of the most prevalent opportunistic infections in patients undergoing immunosuppressive therapy. In this article, we discuss risk factors for PCP development in patients with rheumatoid arthritis (RA) during the course of biologic therapy and describe a prophylactic treatment for PCP with trimethoprim/sulfamethoxazole (TMP/SMX). We also evaluate the effectiveness and safety of the treatment.

**Methods:**

We retrospectively analyzed 702 RA patients who received biologic therapy and compared the characteristics of patients with vs. without PCP to identify the risk factors for PCP. Accordingly, we analyzed 214 patients who received the TMP/SMX biologic agents as prophylaxis against PCP at the start of treatment to evaluate their effectiveness and safety.

**Results:**

We identified the following as risk factors for PCP: age at least 65 years (hazard ratio (HR) = 4.37, 95% confidence interval (CI) = 1.04 to 18.2), coexisting pulmonary disease (HR = 8.13, 95% CI = 1.63 to 40.0), and use of glucocorticoids (HR = 11.4, 95% CI = 1.38 to 90.9). We employed a protocol whereby patients with two or three risk factors for PCP would receive prophylactic treatment. In the study with 214 patients, there were no cases of PCP, and the incidence of PCP was reduced to 0.00 per 100 person-years compared with that before the procedure (0.93 per 100 person-years). There were no severe adverse events induced by the TMP/SMX treatment.

**Conclusions:**

RA patients with two or three risk factors for PCP who are receiving biologic therapy can benefit from safe primary prophylaxis.

## Introduction

A paradigm shift in the treatment of rheumatoid arthritis (RA) has been brought about by the introduction of biologic agents. Special attention should be given to opportunistic infections in RA patients treated with biologics. The strict postmarketing surveillance of an anti-tumor necrosis factor α (anti-TNFα) agent, etanercept (ETN), in Japan showed that 1,206 (8.7%) of 13,894 patients developed infections and 334 patients (2.4%) developed severe infections [1]. Authors of several reports have identified severe infections in patients receiving other biologics and have indicated the significance of prophylaxis for opportunistic infections in RA patients.

*Pneumocystis* pneumonia (PCP) is one of the most prevalent opportunistic infections, and it can lead to potentially lethal respiratory dysfunction in patients with HIV infection. It is also observed in patients with autoimmune diseases undergoing immunosuppressive treatment [2]. Takabayashi *et al*. reported that PCP developed in 9 (1.2%) of 761 patients with autoimmune diseases [3]. It has been demonstrated that PCP in non-HIV patients is more rapidly progressive and fatal than in it is in HIV patients [4]. Several cases of PCP in Crohn’s disease patients who were receiving infliximab (IFX) have been reported [5-7]. Authors of other reports have shown that PCP developed in patients with RA during the course of biologic therapy. Postmarketing surveillance in Japan revealed that 22 (0.44%) of 5,000 patients receiving IFX [8] and 25 (0.18%) of 13,894 patients receiving ETN developed PCP [1].

It is well-known that HIV patients with a CD4+ cell count less than 200 cells/mm^3^ are likely to develop PCP and that the most common identifiable risk factor for developing PCP in patients with autoimmune disease or malignancy is the use of glucocorticoids [9,10]. However, there have been few published reports on the risk factors for PCP development in patients with RA who are receiving biologics, possibly because of the low incidence and small degree of recognition of PCP in Western countries.

In this study, we first retrospectively analyzed patients with RA who had started treatment with biologic agents (TNFα or interleukin 6 (IL-6) inhibitors) before September 2009 to identify the risk factors for PCP development and determined the primary prophylactic procedure. The prophylactic procedure was applied to RA patients who started treatment with biologics between October 2009 and September 2010, then we retrospectively analyzed these cases to estimate the effectiveness and safety of the procedure.

## Methods

### First cohort study to detect risk factors for developing *Pneumocystis* pneumonia

We retrospectively analyzed 702 traceable patients with RA who had started treatment with biologic agents between April 2005 and September 2009 in the First Department of Internal Medicine, University of Occupational and Environmental Health (Fukuoka, Japan). During the first step, we compared the clinical features and laboratory data between patients who developed PCP and the remaining patients who did not receive trimethoprim-sulfamethoxazole (TMP/SMX) prophylaxis. The following clinical parameters were evaluated to identify risk factors for the development of PCP: age, the duration of RA, the Disease Activity Score in 28 joints score and erythrocyte sedimentation rate (DAS28 ESR), coexisting pulmonary disease, diabetes mellitus (DM), ratio of patients who received glucocorticoid treatment, dose of glucocorticoid, dose of methotrexate (MTX), serum immunoglobulin G (IgG) and serum KL-6. Before starting biologic therapy, all patients underwent a bidirectional chest X-ray and/or chest computed tomography (CT) to identify coexisting pulmonary disease, including interstitial pneumonia, pleuritis, diffuse panbronchiolitis, bronchiectasia, old tuberculosis and inflammatory nodules. We excluded the patients who were proven soon after the initiation of biologic agents to have complications due to either malignancy or HIV, which influence the risk for PCP development.

TMP/SMX prophylaxis was begun when each physician considered a patient to be at relatively high risk for PCP based on factors including age, dose of glucocorticoid, serum IgG level, coexisting pulmonary disease and complications of DM.

We compared the characteristics of patients in the PCP group were compared with those in patients without PCP determined risk factors for PCP development. The sensitivity and specificity of the combination of risk factors were used to plan the primary prophylactic procedure, and that procedure was applied to patients with RA who initiated treatment with biologic agents starting in October 2009.

### Second cohort study to estimate effectiveness and safety of prophylaxis

We analyzed 214 patients who initiated biologic therapy between October 2009 and September 2010 to estimate the incidence of PCP, the continuation rate and the adverse events associated with TMP/SMX prophylaxis during the observation period. The observation period was continued after TMP/SMX withdrawal.

### *Pneumocystis* pneumonia prophylaxis

One tablet containing 80 mg of trimethoprim and 400 mg of sulfamethoxazole was given every day, or two tablets were given three times weekly as prophylaxis against PCP, and the doses were tapered in patients with renal dysfunction. TMP/SMX was changed to inhaled aerosolized pentamidine in patients who experienced adverse events due to TMP/SMX treatment. They inhaled aerosolized pentamidine at a dose of 300 mg once monthly.

### Diagnosis of *Pneumocystis* pneumonia

A diagnosis of PCP was presumed when a patient with fever and nonproductive cough had progressive hypoxemia and interstitial pneumonia detected by chest X-ray and/or chest CT and was diagnosed on the basis of positivity for *Pneumocystis jirovecii* in the sputum or bronchoalveolar lavage fluid (BALF) by PCR and/or by elevated concentration of β-D-glucan.

### Statistical analysis

For comparison of the PCP and non-PCP groups, we used the Wilcoxon rank-sum test. Logistic regression analysis was performed to determine risk factors for PCP development by JMP 9.0. A *P* value < 0.05 was taken as statistically significant.

### Ethical considerations

This study is compliant with the Declaration of Helsinki, and the study protocol was approved by the Ethics Committee of the University of Occupational and Environmental Health School of Medicine. Informed consent to participate in the study and have information published was obtained from each patient.

## Results

### First study identified three risk factors for *Pneumocystis* pneumonia development

In the study to develop the prophylactic procedure, we evaluated 702 patients with RA who were being treated with biologic therapy (281 patients treated with IFX, 197 with ETN, 117 with adalimumab (ADA) and 107 with tocilizumab (TCZ)). The patient population comprised 574 females (81.7%) and 128 males (18.3%). The mean age of the patients when they received their first biologic agent was 58.0 years (range, 14 to 86 years) (Table 1). The mean observation period was 16.6 months (range, 2 weeks to 60 months) and 141 patients (20.1%) received TMP/SMX prophylaxis. A total of 265 patients (37.7%) had coexisting pulmonary disease, 303 patients (43.2%) received glucocorticoid therapy and the mean dose (±SD) of glucocorticoid (converted to the prednisolone (PSL) equivalent) was 4.46 mg ± 3.64 mg. We included not only active lung disease but also nonactive lung lesions, including inactive pulmonary fibrosis as coexisting pulmonary disease, which resulted in the high ratio of patients with coexisting lung disease. A total of 78.5% of patients were given MTX, and the average dose of MTX in patients treated with MTX was 8.96 mg/wk.

**Table 1:** **Comparison of baseline characteristics of two cohorts**^**a**^

**Characteristics**	**Before**	**After**	***P*-value**
Patients, *n*	702	214	
Observation period (person-years)	972.9	101.4	
Females, *n* (%)	578 (82.3)	174 (81.3)	0.851
Age (years)	58.0 ± 14.3	58.9 ± 15.5	0.642
RA duration (months)	112 ± 125	92.9 ± 119	0.037
DAS28 score (ESR)	5.83 ± 1.24	5.45 ± 1.27	0.001
Coexisting pulmonary disease (%)	37.7	65.8	0.001
Diabetes mellitus (%)	12.7	9.80	0.268
Glucocorticoids (%)^b^	43.2	22.9	0.001
Methotrexate (%)^c^	78.5	87.9	0.002
Dose of methotrexate (mg/wk)	8.96 ± 2.46	9.51 ± 2.51	0.007
Serum level of IgG (mg/dl)	1590 ± 522	1600 ± 876	0.131
Serum level of KL-6 (U/ml)	271 ± 147	250 ± 194	0.086

^a^DAS28: Disease Activity Score in 28 joints; ESR: Erythrocyte sedimentation rate; IgG: Immunoglobulin G. ^b^The use of glucocorticoids data are the percentage of patients treated with glucocorticoids. ^c^The use of methotrexate data are the percentage of patients treated with methotrexate. Compared with the group of 702 patients, the duration of RA was shorter and DAS28 score (ESR), the complication of pulmonary disease, and the use and dose of glucocorticoids were lower in 214 patients. The dose of methotrexate was increased after the adoption of the prophylaxis criterion. Data are mean ± SD by Wilcoxon rank-sum test.

Nine (1.28%) of the seven hundred two patients developed PCP, and none of the patients in the group treated with TMP/SMX prophylaxis developed PCP. Four of nine patients with PCP were receiving the prophylactic treatment at the initiation of biologic therapy, although all of them stopped the prophylaxis (because of side effects of prophylaxis in two patients and for unknown reasons in two patients). The other five patients were not receiving the prophylaxis during the treatment with biologics. The mean interval between the first infusion of the biologic agent and the onset of PCP was 7.17 months (range, 2 weeks to 20 months). The mean age of patients with PCP at the start of biologic therapy was 69.5 years (range, 57 to 78 years), and the mean morbidity period of RA for the PCP patients was 76.3 months (Table 2). Coexisting pulmonary disease was detected in seven (77.8%) of the nine patients. Eight patients (88.9%) who developed PCP were receiving glucocorticoid therapy at a mean dose (±SD) of 8.83 mg ± 14.9 mg. Six (66.7%) of the nine patients were given MTX, and the average dose of MTX given to them was 9.83 mg/wk. The average in the nine patients was 6.56 mg/wk.

**Table 2:** **Baseline characteristics of the patients who developed *Pneumocystis *** **pneumonia**^**a**^

**Patient**	**Biologic**	**Age (years)**	**Sex**	**RA duration (months)**^**b**^	**Treatment duration (months)**^**c**^	**Pulmonary disease**	**DM**	**DAS28 score (ESR)**	**PSL (mg)**	**KL**-**6 (U/ml)**
1	IFX	71	Female	72	10	(-)	(-)	6.24	2.5	ND
2	IFX	78	Female	79	1	(+)	(-)	6.99	1.0	178
3	IFX	62	Male	21	20	(+)	(-)	7.40	2.0	893
4	IFX	78	Female	40	1	(+)	(-)	8.04	8.0	398
5	IFX	57	Male	5	2	(+)	(-)	3.38	0	304
6	ETN	73	Male	2	3	(+)	(-)	7.20	50	366
7	ETN	59	Female	252	15	(-)	(-)	5.60	1.0	224
8	ETN	75	Male	132	5	(+)	(-)	6.75	5.0	624
9	TCZ	73	Male	84	8	(+)	(-)	7.00	10	445
Mean		69.5		76.3	7.2	7/9	0/9	6.51	8.83	429

^a^PCP developed in nine patients who did receive trimethoprim/sulfamethoxazole prophylaxis. Eight of nine patients were given glucocorticoids. -: Without DM; +: With DM; DAS28: Disease Activity Score in 28 joints; DM: Diabetes mellitus; ESR: Erythrocyte sedimentation rate; ETN: Etanercept; IFX: Infliximab; PCP: *Pneumocystis* pneumonia; PSL: Prednisolone; RA: Rheumatoid arthritis; TCZ: Tocilizumab. ^b^RA duration was measured between the onset of RA and the initiation of biologics. ^c^Treatment duration was measured between the initiation of biologics and PCP development.

Because TMP/SMX is known to inhibit the development of PCP almost completely, we excluded from the analysis the 141 patients treated with the prophylaxis. We first analyzed the characteristics of these 141 patients who were thought to be at high risk for the development of PCP and received the prophylaxis. Compared with the other 561 patients, the mean age in their group was significantly older (68.9 years vs. 55.3 years); the serum level of IgG was significantly lower (1,496 mg/dl vs. 1,619 mg/dl); the dose of MTX was significantly lower (6.09 mg vs. 7.28 mg); and the ratios of patients treated with glucocorticoids, PCP complicated with coexisting pulmonary disease and PCP complicated with DM were significantly higher (53.9% vs. 40.8%, 72.3% vs. 29.1% and 21.3% vs. 10.5%, respectively).

**Figure 1:**
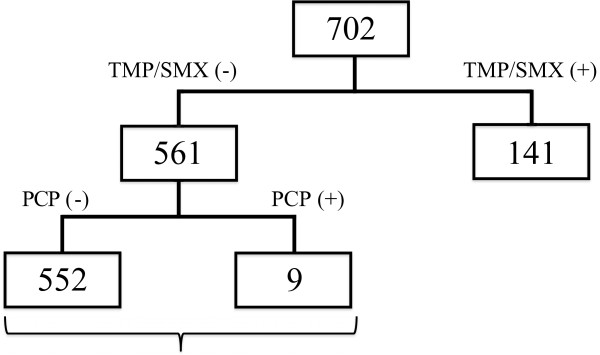
**Schematic of the study protocol.** The 141 patients treated with prophylaxis trimethoprim/sulfamethoxazole (TMP**/**SMX (+)) were excluded from the analysis, and the 561 patients who did not receive prophylaxis (TMP**/**SMX (-)) were analyzed to reveal risk factors for *Pneumocystis* pneumonia (PCP).

**Figure 2:**
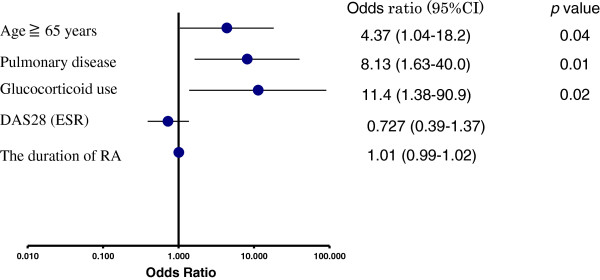
**Graph showing three risk factors for *Pneumocystis *pneumonia.** Logistic regression analysis identified three risk factors: age at least 65 years (hazard ratio (HR) = 4.37, 95% confidence interval (CI) = 1.04 to 18.2), coexisting pulmonary disease (HR = 8.13, 95% CI = 1.63 to 40.0) and the use of glucocorticoids (HR = 11.4, 95% CI = 1.38 to 90.9). The Disease Activity Score in 28 joints (DAS28) score (erythrocyte sedimentation rate (ESR)) and the duration of rheumatoid arthritis (RA) were not risk factors for *Pneumocystis* pneumonia.

Next, we analyzed the clinical and laboratory records of the 561 patients who did not receive TMP/SMX prophylaxis (Figure 1 and Table 3). The patients in the PCP group (*n* = 9) were significantly older, had a higher percentage of coexisting pulmonary disease, had a higher serum level of KL-6 and were treated with a higher PSL dose than the patients in the non-PCP group (*n* = 552). In the PCP group, the percentage of patients treated with glucocorticoids was significantly higher than that in the non-PCP group. There were no significant differences in the duration of morbidity , RA DAS28 score, percentage of patients with DM, dose of MTX or serum IgG level between the PCP and non-PCP groups. Because of the low incidence of PCP, we could not analyze the differences associated with each biologic therapy as a risk factor for PCP. The logistic regression analysis revealed that, among patients with PCP, the hazard ratio (HR) for patients at least 65 years of age was 4.37 (95% confidence interval (CI) = 1.04 to 18.2), the HR for those with coexisting pulmonary disease was 8.13 (95% CI = 1.63 to 40.0) and the HR for use of glucocorticoids was 11.4 (95% CI = 1.38 to 90.9) (Figure 2).

**Table 3:** **Comparison of baseline characteristics of patients with vs. without *Pneumocystis *** **pneumonia in no-prophylaxis group**^**a**^

**Characteristics**	**PCP group**	**Non-PCP group**	***P*-value**
Patients (*n*)	9	552	
Observation period (person-years)	12.5	839.4	
Age (years)	69.5 ± 7.63	57.9 ± 14.1	0.001
Duration of RA (months)	76.3 ± 73.8	112.9 ± 116.5	0.634
DAS28 score (ESR)	6.51 ± 1.28	5.85 ± 1.29	0.060
Coexisting pulmonary disease (%)	77.8	28.1	0.002
Diabetes mellitus (%)	0.00	10.7	0.156
Dose of prednisolone (mg)	8.83 ± 14.9	1.70 ± 2.57	0.004
Glucocorticoids (%)^b^	88.9	39.9	0.009
Dose of methotrexate (mg/w)	6.56 ± 5.23	7.29 ± 4.10	0.695
Serum level of IgG (mg/dl)	1,600 ± 714	1,620 ± 511	0.660
Serum level of KL-6 (U/ml)	429 ± 218	257 ± 138	0.008

^a^DAS28: Disease Activity Score in 28 joints; ESR: Erythrocyte sedimentation rate; IgG: immunoglobulin G; PCP: *Pneumocystis* pneumonia; RA: Rheumatoid arthritis. ^b^Glucocorticoid data are percentages of patients treated with glucocorticoids.

Patient age, the complication of pulmonary disease, the use and dose of glucocorticoids and the serum level of KL-6 were significantly different between the two groups. Data are mean ± SD by Wilcoxon rank-sum test unless otherwise specified.

We calculated the sensitivity and specificity of these risk factors as tests to represent the incidence of PCP. The group of patients with one or more risk factors for PCP includes all patients with PCP, in addition to many patients without PCP (sensitivity = 100%, specificity = 34.1%). If prophylaxis were given only to patients with three risk factors, we would have missed four of nine patients with PCP (sensitivity = 55.6%, specificity = 94.4%). On the basis of these results, we assumed that patients with two or three risk factors could benefit from prophylaxis with TMP/SMX (sensitivity = 77.8%, specificity = 76.1%). Assuming that TMP/SMX inhibits the development of PCP completely, the number needed to treat to prevent one case of PCP was 19.9 in the analysis of 561 patients.

### Second study demonstrated effectiveness of prophylaxis protocol

The inclusion criteria for PCP prophylaxis were applied for 214 patients with RA for whom treatment with biologic agents was initiated between October 2009 and September 2010. The mean age of the 214 patients (67 patients treated with IFX, 51 with ETN, 64 with ADA and 32 with TCZ) was calculated for 174 females (81.3%) and 40 males (18.7%). The mean age in this group was 58.9 years (range = 15 to 89 years). The mean observation period was 5.7 months. The mean (±SD) DAS28 (ESR) score was 5.45 ± 1.27, and the mean duration of RA was 92.9 ± 119 months. A total of 94 patients (43.9%) received prophylaxis against PCP. Of the 214 patients, 141 (65.8%) had coexisting pulmonary disease and 49 (22.9%) received glucocorticoid therapy, and the mean glucocorticoid dose (converted to the PSL equivalent) was 6.28 mg ± 6.46 mg. In this group of 214 patients, 87.9% were given MTX and the average dose of MTX was 9.51 mg/wk.

Among the primary prophylactic group (94 patients), 12 patients (12.8%) received prophylaxis with inhaled aerosolized pentamidine starting from the initiation period for the following reasons: four patients had renal dysfunction, four had cytopenia and four had other conditions. Among the patients who received prophylaxis with TMP/SMX (82 patients), adverse events occurred in 11 patients (13.4%). These side effects included liver dysfunction (five patients), cytopenia (two patients), skin eruption (one patient), renal dysfunction (one patient), hyponatremia (one patient) and an unspecified other adverse event (one patient). TMP/SMX prophylaxis was changed to inhaled aerosolized pentamidine in all 11 of these patients. There were no severe adverse events that required hospital admission. The observation period was continued after TMP/SMX withdrawal.

It is noteworthy that, during the observation period, no case of PCP was observed among the patients in the second study. Compared with the results of the first analysis of 702 patients, the incidence of PCP decreased from 1.28% (9 of 702 patients) and 0.93/100 patient-years to 0.00% and 0/100 patient years, which confirms the effectiveness of the prophylactic procedure.

## Discussion

It is well-known that HIV patients with a CD4+ cell counts less than 200 cells/mm^3^ are likely to develop PCP, and the most common identifiable risk factor for developing PCP in patients with autoimmune disease or malignancy is glucocorticoid use [9,10]. Ogawa *et al*. reported that the number of peripheral lymphocytes at 2 weeks after initiation of glucocorticoid treatment, not the number at the initiation of treatment, was a risk factor for PCP in patients with rheumatic diseases [11]. Other reports and some postmarketing surveillance studies have revealed that a low lymphocyte count is not a risk factor for PCP in patients with RA. Thus, we excluded the number of lymphocytes as a risk factor from the analysis.

The postmarketing surveillance of IFX revealed that the development of PCP in RA patients treated with IFX was best predicted by an age of at least 65 years, dose of glucocorticoids (≥6 mg of PSL) and coexisting pulmonary disease [12]. However, that report was restricted to patients treated with IFX and did not include patients receiving other TNFα inhibitors or an IL-6 inhibitor. After analyzing the patients treated with biologic therapy who developed PCP in this study, we found that four of nine patients had fewer than two of the above-mentioned risk factors.

In our present study, we identified various risk factors for PCP in patients treated with biologics, including not only TNFα inhibitors but also the IL-6 inhibitor, to establish a new and useful prophylactic TMP/SMX procedure for PCP. We retrospectively compared a PCP group with a non-PCP group in patients who did not receive prophylaxis, and the multivariate analysis revealed that an age of at least 65 years, coexisting pulmonary disease and use of glucocorticoids were risk factors for PCP. An age of at least 65 years and coexisting pulmonary disease were also risk factors in the IFX postmarketing surveillance report; however, in our present study, the use of glucocorticoids alone was abstracted as a risk factor without regard to the dose administered. Other researchers have shown that, among patients with interstitial pneumonia or autoimmune disease, the glucocorticoid dose (≥30 mg of PSL) was a risk factor for PCP development [3,11]. In our study, eight of the nine patients who developed PCP were receiving glucocorticoids, but only two patients were given at least 6 mg of PSL. The other six patients were treated with less than 6 mg of PSL (1.0 mg to 5.0 mg), suggesting that patients treated with glucocorticoids can benefit from primary TMP/SMX prophylaxis of PCP, regardless of glucocorticoid dose.

A previous report revealed that patients treated with high-dose glucocorticoids, those receiving immunosuppressive agents and those with lower serum IgG levels had a significantly higher risk for developing PCP [13]. Another recent report identified an advanced radiographic stage as a risk factor for PCP in RA patients treated with ADA [14]. In the present study, however, there were no significant differences in the serum IgG level or the radiographic stage between the patients with versus without PCP. Although concomitant MTX treatment was identified as an independent risk factor for PCP in a report of RA patients in Japan who were treated with ETN [15], the use (yes or no) or dose of MTX was not found to be a risk factor for the development of PCP in our patient cohort.

The duration between the initiation of biologics and PCP development varied widely, from 2 weeks to 20 months. In previous reports of RA patients treated with biologics, 76% to 90% of the cases of PCP developed within 6 months after the initiation of biologics [14-16]. In our present study, PCP developed in two of nine patients more than 12 months after biologics were started. That finding suggests that TMP/SMX prophylaxis needs to be continued for long periods.

A total of 250 (35.6%) of the 702 patients had two or three risk factors for PCP in this study. If patients with at least one risk factor were started on TMP/SMX prophylaxis in our study, 513 (73.1%) of the patients would have been included in the prophylaxis group. We thought that including these patients would have been difficult because of the adverse effects of the prophylactic treatment.

In the analysis of 214 patients, the duration of RA was shorter and the DAS28 score (ESR) was lower than those of the 702 patients. These findings could be due to our becoming more proactive about using biologic agents in patients with RA during the early stage of the disease. After adoption of the inclusion criteria for PCP prophylaxis, the ratio of glucocorticoid use decreased and the MTX dose increased, suggesting that an adequate dosage of MTX led to a tendency toward decreased glucocorticoid use in patients with RA. Almost all of the patients underwent CT, and we were more proactive about using biologic agents in patients with complications after the adoption of the prophylactic criteria. This is why the percentage of pulmonary disease was much higher in the 214 patients than that in the analysis of 702 patients.

Under the new prophylactic criteria, 94 (43.9%) of 214 patients were given prophylaxis and no severe adverse events occurred. After the adoption of the criteria for PCP prophylaxis, none of the patients developed PCP and the incidence of PCP was reduced from 0.93/100 patient-years to 0.00, confirming the validity and the safety of the primary prophylactic procedure in daily clinical practice. Authors of a previous report found that 3.1% of patients were forced to stop TMP/SMX prophylaxis because of adverse events [17]. The development of adverse events following TMP/SMX treatment were unavoidable in our present study, but we found that inhaled aerosolized pentamidine could be used as an alternative prophylactic treatment, rather than biologics, for PCP in patients with RA.

There are some limitations associated with this study. First, we treated only nine patients with PCP A larger number of patients and a longer observation period are necessary to confirm the effectiveness of our protocol. By the statistical calculation, we found that we needed 369 patients to confirm the statistical significance of the difference in the incidence of PCP in the first cohort (1.28%). Second, *Pneumocystis* colonization, which was previously reported to be a possible risk factor for PCP, was not analyzed in this study. The number of lymphocytes also was not analyzed. In the first cohort evaluated to identify the risk factors for PCP development, we excluded 141 patients who received TMP/SMX, although this exclusion might have led to biased results regarding the identification of risk factors. The serum level of IgG and the ratio of patients with DM complications were significantly different between the excluded 141 patients and 561 patients, respectively. These results suggest that if all 141 patients had not received the prophylaxis and the incidence of PCP had been much higher than 1.28%, the lower IgG level or the complication with DM might have been risk factors for PCP.

The incidence of PCP is much lower in Western countries than in Japan. Given the adverse effects of TMP/SMX, the prophylaxis criteria used in this study cannot be applied to patients in other countries in which the incidence of PCP is quite different from that in Japan. Additional studies are required to determine the incidence and risk factors for PCP in Western countries.

## Conclusions

The results of the first analysis in this study show that there are three major risk factors for the development of PCP in patients with RA receiving biologics. They reveal that patients with two or three risk factors could benefit from TMP/SMX prophylaxis against PCP. We also show the prophylactic effectiveness and safety of the inclusion criteria. In patients 65 years of age or older, coexisting pulmonary disease and the use of glucocorticoids were identified as risk factors for PCP in RA patients being treated with biologics. Patients with two or three risk factors are therefore considered to benefit from primary prophylaxis against PCP. However, the number of patients was too low, so this prophylactic procedure needs to continue to be applied to further investigate its validity and safety.

## Abbreviations

ADA: Adalimumab; BALF: Bronchoalveolar lavage fluid; CT: Computed tomography; DAS: Disease Activity Score; ETN: Etanercept; IFX: Infliximab; IL: Interleukin; MTX: Methotrexate; PCP: *Pneumocystis* pneumonia; PSL: Prednisolone; RA: Rheumatoid arthritis; TCZ: Tocilizumab; TMP/SMX: Trimethoprim/sulfamethoxazole; TNFα: Tumor necrosis factor α.

## Competing interests

YT has received consulting fees, speaking fees and/or honoraria from Chugai Pharma, Mitsubishi-Tanabe Pharma, Eisai Pharma, Pfizer, Abbott Immunology Pharma, Janssen Pharma, Takeda Industrial Pharma, Astra-Zeneca, Astellas Pharma, Asahi-kasei Pharma and GlaxoSmithKline, and he has received research grant support from Bristol-Myers Squibb, Mitsubishi-Tanabe Pharma, MSD, Takeda Industrial Pharma, Astellas Pharma, Eisai Pharma, Chugai Pharma, Pfizer and Daiichi-Sankyo.

## Authors’ contributions

TK acquired, analyzed and interpreted data; performed the statistical analysis; and wrote and revised the manuscript. SK and MN acquired, analyzed and interpreted data; performed the statistical analysis; and drafted the manuscript. KS interpreted data and edited and revised the manuscript. YT designed the study, interpreted data and edited the manuscript. All authors read and approved the final manuscript.
